# Experience in a Climate Microworld: Influence of Surface and Structure Learning, Problem Difficulty, and Decision Aids in Reducing Stock-Flow Misconceptions

**DOI:** 10.3389/fpsyg.2018.00299

**Published:** 2018-03-26

**Authors:** Medha Kumar, Varun Dutt

**Affiliations:** ^1^Applied Cognitive Science Laboratory, School of Computing and Electrical Engineering, Indian Institute of Technology Mandi, Kamand, India; ^2^School of Humanities and Social Sciences, Indian Institute of Technology Mandi, Kamand, India

**Keywords:** stock-and-flow simulations, correlation heuristic, violation of mass balance, experience, problem structure, decision aids, heterogeneity, Dynamic Climate Change Simulator

## Abstract

Research shows that people’s wait-and-see preferences for actions against climate change are a result of several factors, including cognitive misconceptions. The use of simulation tools could help reduce these misconceptions concerning Earth’s climate. However, it is still unclear whether the learning in these tools is of the problem’s surface features (dimensions of emissions and absorptions and cover-story used) or of the problem’s structural features (how emissions and absorptions cause a change in CO_2_ concentration under different CO_2_ concentration scenarios). Also, little is known on how problem’s difficulty in these tools (the shape of CO_2_ concentration trajectory), as well as the use of these tools as a decision aid influences performance. The primary objective of this paper was to investigate how learning about Earth’s climate via simulation tools is influenced by problem’s surface and structural features, problem’s difficulty, and decision aids. In experiment 1, we tested the influence of problem’s surface and structural features in a simulation called Dynamic Climate Change Simulator (DCCS) on subsequent performance in a paper-and-pencil Climate Stabilization (CS) task (*N* = 100 across four between-subject conditions). In experiment 2, we tested the effects of problem’s difficulty in DCCS on subsequent performance in the CS task (*N* = 90 across three between-subject conditions). In experiment 3, we tested the influence of DCCS as a decision aid on subsequent performance in the CS task (*N* = 60 across two between-subject conditions). Results revealed a significant reduction in people’s misconceptions in the CS task after performing in DCCS compared to when performing in CS task in the absence of DCCS. The decrease in misconceptions in the CS task was similar for both problems’ surface and structural features, showing both structure and surface learning in DCCS. However, the proportion of misconceptions was similar across both simple and difficult problems, indicating the role of cognitive load to hamper learning. Finally, misconceptions were reduced when DCCS was used as a decision aid. Overall, these results highlight the role of simulation tools in alleviating climate misconceptions. We discuss the implication of using simulation tools for climate education and policymaking.

## Introduction

Understanding stocks and flows is a fundamental process in the real world (Dörner, 1996; Sterman, 2008, 2011; Cronin et al., 2009; Fischer et al., 2015). For example, we maintain our bank accounts (a stock) as a result of our incomes (inflows) and expenses (outflows); we support our body weight (a stock) by managing our diet (inflow) and exercise (outflow); and, we maintain carbon-dioxide levels in the atmosphere (a stock) by emissions (inflow) and absorption (outflow) (Cronin et al., 2009; Dutt, 2011). Different stock-flow problems share the same underlying structure: A stock or level accumulates the inflows to it less the outflows from it (Sweeney and Sterman, 2000).

It is a well-known phenomenon that people have difficulties in understanding the dynamics of stock-flow problems (Dörner, 1996; Sterman, 2008, 2011; Cronin et al., 2009; Dutt, 2011). Stock-flow problems, even simple ones involving one stock and two flows (inflow and outflow), are difficult, even for highly educated people with strong mathematics backgrounds (Sweeney and Sterman, 2000; Sterman and Sweeney, 2002; Sterman, 2008, 2011; Cronin et al., 2009; Dutt, 2011). For example, Sweeney and Sterman (2000) presented graduate students at Massachusetts Institute of Technology with a picture of a bathtub and graphs showing the inflow and outflow of water, then asked them to sketch the trajectory of the stock of water in the tub. Although the patterns were simple, fewer than half responded correctly. We denote such difficulties in responding to stock-flow failure.

Stock-flow failure has also been documented in problems concerning Earth’s climate system (Dutt, 2011). Here, people find it difficult to sketch the shape of emissions and absorptions corresponding to a carbon-dioxide (CO_2_) concentration trajectory. Two of the prevalent misconceptions in climate stock-flow problems are the correlation heuristic and violation of mass balance (Dutt and Gonzalez, 2012a,b). According to the correlation heuristic, people incorrectly infer that an accumulation (CO_2_ concentration) follows the same path as the inflow (CO_2_ emissions). This misconception assumes that stabilizing emissions would rapidly stabilize the concentration; and, emission cuts would quickly reduce the concentration and damages from climate change. This reasoning is incorrect because reliance on the correlation heuristic significantly underestimates the time delays existent between reductions in CO_2_ emissions and their effect on the CO_2_ concentration (Sterman, 2008; Dutt and Gonzalez, 2012a, 2013a,b; Kumar and Dutt, unpublished).

According to the second misconception in climate stock-flow problems, violation of mass balance, people incorrectly infer that atmospheric CO_2_ concentration can be stabilized even when emissions exceed absorptions. According to mass balance violation, people think that the current state of the Earth’s climate, where emissions are about double that of absorptions, would not pose a problem to future stabilization (Sterman, 2008; Dutt and Gonzalez, 2012a; Kumar and Dutt, unpublished).

Although people’s wait-and-see preferences for actions against climate change are a result of several factors like social identities, party-affiliations, and denial (McCright and Dunlap, 2011), recent research has shown that climate misconceptions are also likely to influence such preferences (Dutt, 2011). Specifically, correlation heuristic thinking leads to wait-and-see preferences because people believe that stabilizing CO_2_ emissions is sufficient to stabilize the CO_2_ concentration. Similarly, violation of mass balance thinking leads to wait-and-see choices because people believe that CO_2_ concentration can be stabilized even when CO_2_ emissions are double that of absorptions (Sterman, 2008; Dutt and Gonzalez, 2012a; Kumar and Dutt, unpublished).

Prior research has used a Climate Stabilization (CS) task to test for correlation heuristic and violation of mass balance misconceptions (Sterman and Sweeney, 2007; Sterman, 2008; Dutt and Gonzalez, 2012a,b). In the CS task, participants are given the concentration’s starting value in the year 2000 and its historical trend between 1900 and 2000 on paper. Participants are asked to sketch the CO_2_ emissions and absorptions shapes that would correspond to the projected scenario of CO_2_ concentration between 2001 and 2100. Irrespective of educational backgrounds, people show widespread reliance on correlation heuristic and committing of violation of mass balance in their sketches in the CS task (Sterman and Sweeney, 2007; Sterman, 2008; Dutt and Gonzalez, 2012a). Overall, the CS task has been used as a measure for assessing people’s stock-flow misconceptions concerning climate change (Sterman, 2008; Fischer et al., 2015).

Furthermore, recent research has documented the role that repeated feedback about cause-and-effect relationships plays on human understanding of dynamic systems, particularly for Earth’s climate system (Moxnes and Saysel, 2009; Dutt and Gonzalez, 2012a). Researchers have used computer-based simulation tools and decision-making games (called microworlds) to provide repeated feedback, where a reduction in people’s correlation heuristic and violation of mass balance misconceptions has been demonstrated regarding Earth’s climate system (Dutt and Gonzalez, 2012a, 2013b; Kumar and Dutt, unpublished) and, dynamic systems more generally (Gonzalez et al., 2005; Gonzalez and Dutt, 2011; Dutt and Gonzalez, 2012b). For example, Dutt and Gonzalez (2012a) made participants perform in a Dynamic Climate Change Simulator (DCCS) microworld and then transferred them to the CS task immediately. Participants controlled CO_2_ concentration to a goal level in DCCS by deciding the CO_2_ emissions and absorptions. Next, in the CS task, participants sketched the CO_2_ emissions and absorptions corresponding to a CO_2_ concentration stabilization trajectory. Results revealed that exposure to DCCS before CS task reduced correlation heuristic and violation of mass balance misconceptions.

Although prior research has documented a reduction in correlation heuristic and violation of mass balance due to exposure to simulation tools, little is known on how people improve their stock-flow misconceptions when they interact with these tools. For example, Dutt and Gonzalez (2012a) gave their participants the same problem in DCCS as well as the following CS task. As the problem did not change between DCCS and CS task, it is unclear whether people learnt the structural features (how emissions and absorptions cause a change in CO_2_ concentration under different CO_2_ concentration scenarios) or the surface features (dimensions of emissions, absorptions, and concentration; and, the cover-story used) of the problem in DCCS before attempting the CS task.

While performing in DCCS, one possibility is that people may learn the problem’s structural features. For example, recent research has shown that structural knowledge helps people reduce their correlation heuristic and violation of mass balance misconceptions in both cases when problems encountered in the CS task are structurally similar or different compared to those presented in DCCS (Kumar and Dutt, unpublished). However, while performing in DCCS, another possibility is that people learn the surface features of the climate problem (Chi et al., 1981; Gonzalez and Wong, 2012).

In literature, procedural reinstatement principle states that performance would be better at transfer when the problems encountered during transfer are similar to those encountered during training (Healy et al., 2005). Also, heterogeneity of practice hypothesis states that training on heterogeneous (diverse) problems improves performance during transfer (Gonzalez and Madhavan, 2011). Because of the procedural reinstatement principle (Healy et al., 2005), we expect better performance when problems in the CS task (transfer) are similar in structural features or surface features to those that are learned during DCCS training (before the CS task). Also, because of heterogeneity of practice hypothesis (Gonzalez and Madhavan, 2011), we expect problems with surface or structural training during DCCS would likely produce a more efficient transfer of knowledge and improved performance in the CS task.

Moreover, as per the difficulty hypothesis, people’s transfer of learning is improved when they train on difficult problems compared to easy problems (Schneider et al., 2002; Healy et al., 2005; Young et al., 2011). Thus, if people are subjected to difficult problems in DCCS, then they would likely be able to reduce their correlation heuristic and violation of mass balance misconceptions in the CS task due to the effects stated in the difficulty hypothesis. One way to create difficulty of problems in DCCS is by changing the shape of the CO_2_ concentration curve presented: If the shape of the concentration curve is curvilinear, then this curvilinear shape would create more perceived difficulty among participants compared to when the concentration curve is straighter.

However, it is also possible that a difficult curve in DCCS may not help reduce correlation heuristic and violation of mass balance misconceptions in the CS task because of the predictions of the cognitive load theory (Sweller, 1994; De Jong, 2010). According to cognitive load theory, people possess bounded working memory capacity (Simon, 1959). Thus, if a learning task requires too much-working memory capacity, learning may get hampered (De Jong, 2010). In the DCCS task, it is possible that the processing of different elements like emission, absorption, and concentration requires certain working memory capacity. Also, the processing of the curvilinear CO_2_ concentration curve shape may further need additional working memory capacity. Due to the overload of working memory capacity, participants may not be able to learn the stock-flow relationships in DCCS and reduce their correlation heuristic and violation of mass balance misconceptions in the CS task.

Finally, simulation tools could also be used as a side-by-side decision aid that helps people understand relationships between emissions, absorptions, and concentration by a trial-and-error procedure. There is evidence that even in simple descriptive binary-choice decision tasks, when participants are provided with experiential decision aids, they tend to rely on the experience gained in these aids in making descriptive decisions and improve their decision making (Jessup et al., 2008; Camilleri and Newell, 2011; Lejarraga and Gonzalez, 2011). When DCCS is given as aid, people are likely to get a chance to try different emissions and absorptions and see their effect on concentration. Thus, misconceptions are possible to reduce significantly when people are given an opportunity to try different values of emissions and absorptions in DCCS and to test their effect on the shape of the concentration trajectory.

The primary goal of this research is to investigate via lab-based experiments people’s stock-flow misconceptions about climate change and the role that different factors like surface and structural features, problem difficulty, and decision aids play in reducing people’s stock-flow misconceptions. Such research may help policymakers formulate appropriate policies for climate education in schools and colleges that make use of simulation tools to supplement conventional teaching (Meadows et al., 2016). Furthermore, this research would help provide theoretical and practical advancements in understanding the effectiveness of repeated feedback through simulation tools as an intervention in reducing misconceptions.

In what follows, we first present the background where we highlight prior research and motivate our hypotheses. Next, we report three experiments where we test how problem’s surface and structural features, problem’s difficulty, and decision aids help reduce misconceptions about climate change. In the first experiment, we present how problems with surface or structural training during DCCS help reduce misconceptions in the CS task. In the second experiment, we investigate how problem difficulty during DCCS training help reduce misconceptions in the CS task. In the final experiment, we study how DCCS as a decision aid helps in lowering correlation heuristic and violation of mass balance misconceptions by allowing participants to test different values of emissions and absorptions in a trial-and-error procedure. We close the paper by discussing our results and highlighting the implications of using simulation tools (like DCCS) in education and policymaking against climate change.

## Background Section

**FIGURE 1:**
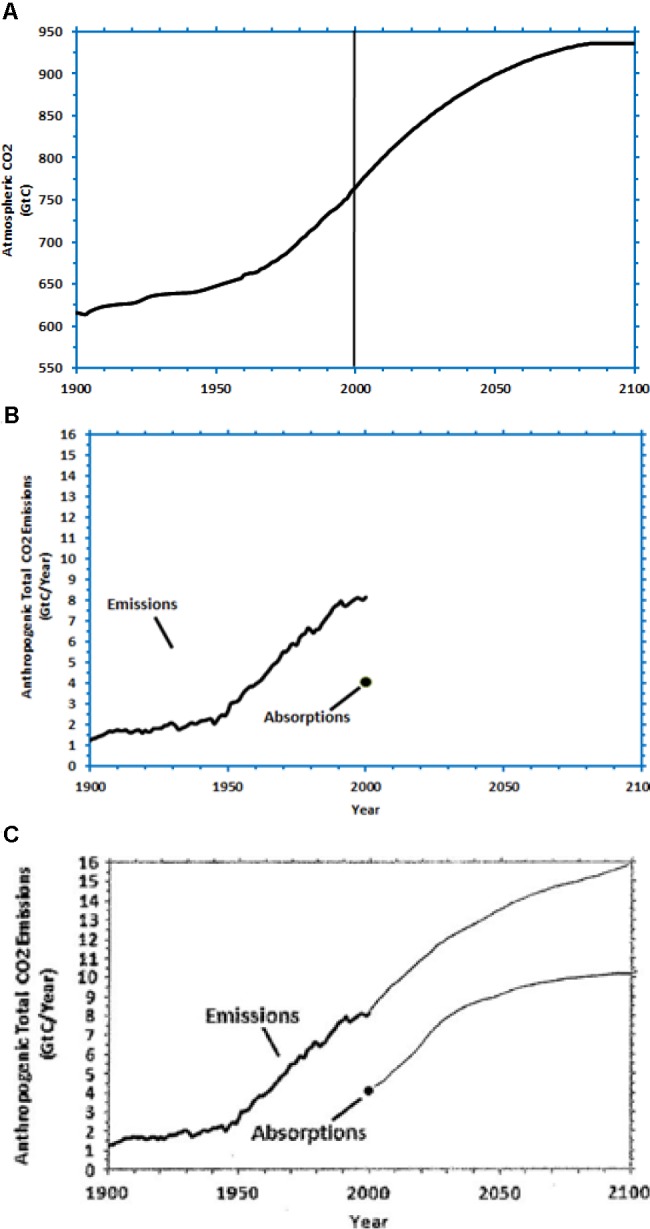
The Climate Stabilization (CS) task. Participants are given CO_2_ concentration stabilization scenario, and they are required to sketch the CO_2_ emissions and absorptions corresponding to the scenario. **(A)** The problem presented shows increasing trajectory where CO_2_ increases and stabilizes by 2100. **(B)** Values of emission and absorption between year 1900 and 2000. **(C)** A typical sketch by participants in the CS task relying on correlation heuristic and violation of mass balance for the increasing trajectory (Source: Dutt and Gonzalez, 2012a).

Prior research in stock-flow problems concerning Earth’s climate has analyzed reliance on correlation heuristic and violation of mass balance in the CS task (Sterman and Sweeney, 2007; Sterman, 2008; Dutt and Gonzalez, 2012a) (see **Figure 1**). In the CS task, participants are asked to sketch CO_2_ emissions and absorptions that would stabilize the CO_2_ concentration according to a given scenario by the year 2100 (given in **Figure 1A**). Participants are given the concentration’s starting value in the year 2000 (**Figure 1B**), and its historic trends and emissions between the years 1900 and 2000. Participants are asked to sketch the CO_2_ emissions and absorptions shapes that would correspond to the projected scenario of CO_2_ concentration between 2001 and 2100. **Figure 1C** shows an example of a participant that relied on correlation heuristic, whereby he inferred that the shapes of the CO_2_ emissions and concentration should look alike. Moreover, as seen in **Figure 1C**, the participant commits violation of mass balance in her response as she fails to make emissions equal to absorption when the concentration reaches 2100. This paper uses the CS task with different CO_2_ concentration trajectories and cover stories to evaluate people’s reliance on correlation heuristic and violation of mass balance misconceptions.

**FIGURE 2:**
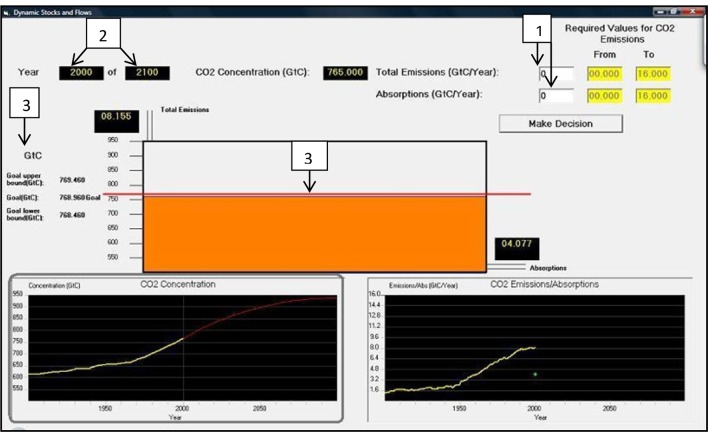
The Dynamic Climate Change Simulator (DCCS) task. DCCS is a dynamic replica of the CS task. (1) Participants set yearly CO_2_ emissions and absorptions and press “Make Decision” button. (2) The system now moves forward a certain number of years. (3) Participants need to maintain their CO_2_ concentration at the red goal line in the tank (which represents the atmosphere) and follow the CO_2_ concentration trajectory shown in the bottom left panel (Source: Dutt and Gonzalez, 2012a).

Furthermore, recent research has evaluated how repeated feedback in DCCS helps reduce correlation heuristic and violation of mass balance misconceptions (Moxnes and Saysel, 2009; Dutt and Gonzalez, 2012a,b; Kumar and Dutt, unpublished). As shown in **Figure 2**, DCCS is a dynamic replica of the CS task, it is based on a simplified and adapted climate model (Dutt and Gonzalez, 2012b), and it has been inspired by generic dynamic stocks-and-flows tasks (Gonzalez et al., 2005; Gonzalez and Dutt, 2011). In DCCS participants set yearly CO_2_ emissions and absorptions and press “Make Decision” button. Upon pressing the “Make Decision” button, the system moves forward a certain number of years. Participants need to maintain their CO_2_ concentration at the red goal line in the tank (which represents the atmosphere) and follow the CO_2_ concentration trajectory shown in the bottom left panel.

Although DCCS helps reduce people’s misconceptions compared to a no-DCCS intervention (Dutt and Gonzalez, 2012a); however, little is currently known on how this reduction is influenced by problem’s surface and structural features, problem’s difficulty, and use of decision aids. The goal of this paper is to investigate the role of these factors in reducing people’s misconceptions concerning the climate system.

First, we propose to create heterogeneous problems during DCCS training and transfer participants from DCCS training to similar/different problems in the CS task. The similarity or differences in problems between training and transfer will allow us to test participants’ surface or structural learning. According to the heterogeneity of practice hypothesis (Gonzalez and Madhavan, 2011), we expect problems with surface or structural training during DCCS training to likely produce more effective transfer of knowledge and improved performance in the following CS task. Also, because of the procedural reinstatement principle (Healy et al., 2005), we expect better performance when problems in the CS task are similar in structure or surface features to those that are learned during DCCS training.

Moreover, if people are subjected to difficult problems in DCCS, then they would likely be able to reduce their correlation heuristic and violation of mass balance misconceptions in the CS task due to the difficulty hypothesis (Schneider et al., 2002; Healy et al., 2005; Young et al., 2011). However, on account of cognitive load theory and people’s bounded working memory capacity (Simon, 1959; Sweller, 1994; De Jong, 2010), it is also likely that if people are subjected to difficult problems in DCCS, then they would not be able to reduce their correlation heuristic and violation of mass balance misconceptions in the CS task.

Another factor that is likely to influence people’s misconceptions about climate system is the use of simulation tools as decision aids (Jessup et al., 2008; Camilleri and Newell, 2011; Lejarraga and Gonzalez, 2011). Thus, providing an experiential DCCS decision aid side-by-side to the CS task is likely to improve decision making in the CS task compared to a condition without the decision aid. In the next section, we detail experiments where we evaluated the influence of problem’s surface and structural features, problem’s difficulty, and use of decision aids on people correlation heuristic and violation of mass balance misconceptions.

## Experiment 1: Influence of Surface and Structural Features in Reducing Stock-Flow Misconceptions

In the first experiment, we test the influence of learning of surface and structural features in DCCS for reducing people’s misconceptions against climate change. Here, we will train people on heterogeneous problems in DCCS, which are diverse in surface and structural features. According to the heterogeneity of practice hypothesis (Gonzalez and Madhavan, 2011), one expects problems with surface or structural training during DCCS would likely produce more effective transfer of knowledge and improved performance in the CS task.

### Methods

#### Participants

Participants were recruited through an email advertisement for a climate study at Indian Institute of Technology Mandi, India. This study was carried out in accordance with the recommendations of Ethics Committee at Indian Institute of Technology Mandi with a written informed consent from all participants. Participation was voluntary and all participants gave written informed consent before starting their study. There were 100 participants in all (74 males and 26 females). Ages ranged from 18 to 26 years (average = 21 years; *SD* = 1.5 years). All participants were students from Science, Technology, Engineering, and Mathematics backgrounds (73% undergraduate, 19% masters, and 8% doctoral). They were randomly assigned to one of the experimental conditions involving DCCS and CS tasks. Participants were paid a flat fee of INR 50 (approximately 0.9 USD) for their participation after they completed the study.

#### Experimental Design

Participants were randomly assigned to one of four between-subjects conditions (*N* = 25^1^ in each condition): CS-Surface, CS-Structure, DCCS-Surface, and DCCS-Structure. In both DCCS-Surface and DCCS-Structure conditions, participants played 2-rounds of DCCS repeatedly with heterogeneous problems that were either based upon surface features or structural features and were then transferred to the CS task immediately. In the CS-Surface and CS-Structure conditions, participants played an unrelated task for the average time it took to complete 2-rounds in DCCS and they were then transferred to the CS task immediately. Heterogeneity in problems was either based upon surface features or structural features.

**FIGURE 3:**
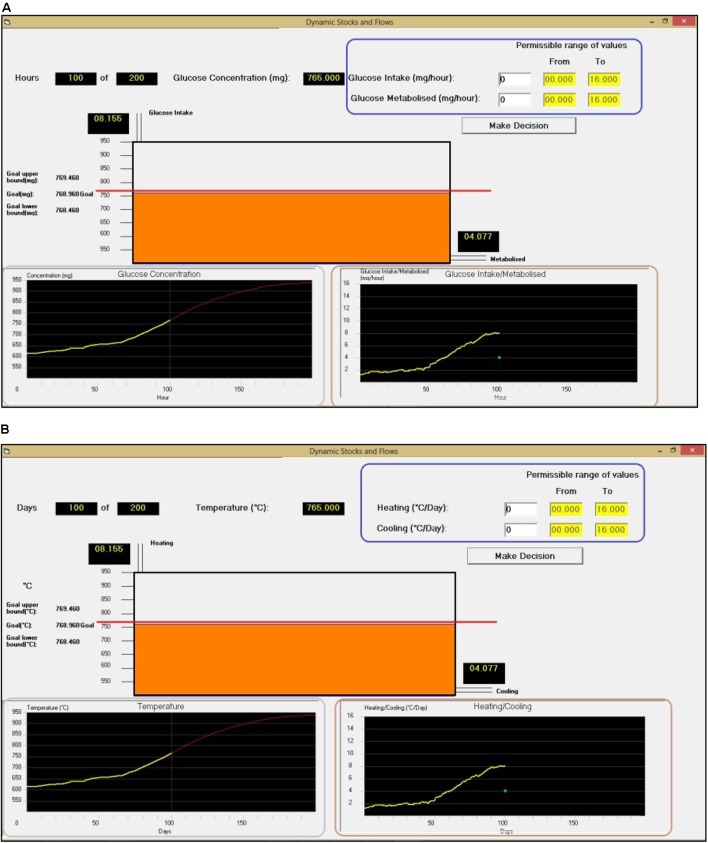
Dynamic Climate Change Simulator tasks in the DCCS-Surface condition where participants need to decide the inflow and outflow values every 5 time periods such that the accumulation (Glucose Concentration or Temperature) followed the red trajectory in the bottom-left Figure. **(A)** DCCS with the glucose cover story (DCCS-Gluc). **(B)** DCCS with the temperature cover story (DCCS-Temp).

Surface features refer to the dimensions of emissions, absorptions, and concentration; and, the cover-story used in DCCS. In the DCCS-Surface condition, participants first tackled **Figure 1**’s problem in each of the two rounds repeatedly in DCCS, however, the problem presented in each round differed randomly in the cover story and units used (i.e., in surface features). As shown in **Figure 3**, we used a glucose cover story (DCCS-Gluc; see **Figure 3A**; inflow = glucose intake, outflow = glucose metabolized, and accumulation = glucose concentration in blood over 100 time periods) and a temperature cover story (DCCS-Temp; see **Figure 3B**; inflow = heating, outflow = cooling and accumulation = temperature in a room over 100 time periods). In each of these two problems, participants controlled their accumulation trajectory in DCCS along a stabilization trajectory by making inflow and outflow decisions every 5 time periods repeatedly. After finishing two rounds in DCCS, participants were transferred to the CS task where they attempted two problems that were presented in a random order. Both these problems corresponded to **Figure 1**’s problem, where one of the problems was presented with the climate cover story (CS-Climate; i.e., just like **Figure 1**’s problem and different from problems presented during DCCS training), while the other problem was presented with the temperature cover story (CS-Temp; i.e., similar to one of the problems during the DCCS training). In both problems, participants needed to sketch the shape of inflow and outflow that corresponded to the accumulation stabilization scenario. The CS-Surface condition contained the same two problems as part of the CS task in the DCCS-Surface condition; however, the CS-Surface condition did not include DCCS training prior to the CS task. In the CS-Surface condition, participants played an unrelated Tetris game before performing in CS tasks for a duration that equaled the time taken to finish 2-rounds of DCCS performance in the DCCS-Surface condition.

**FIGURE 4:**
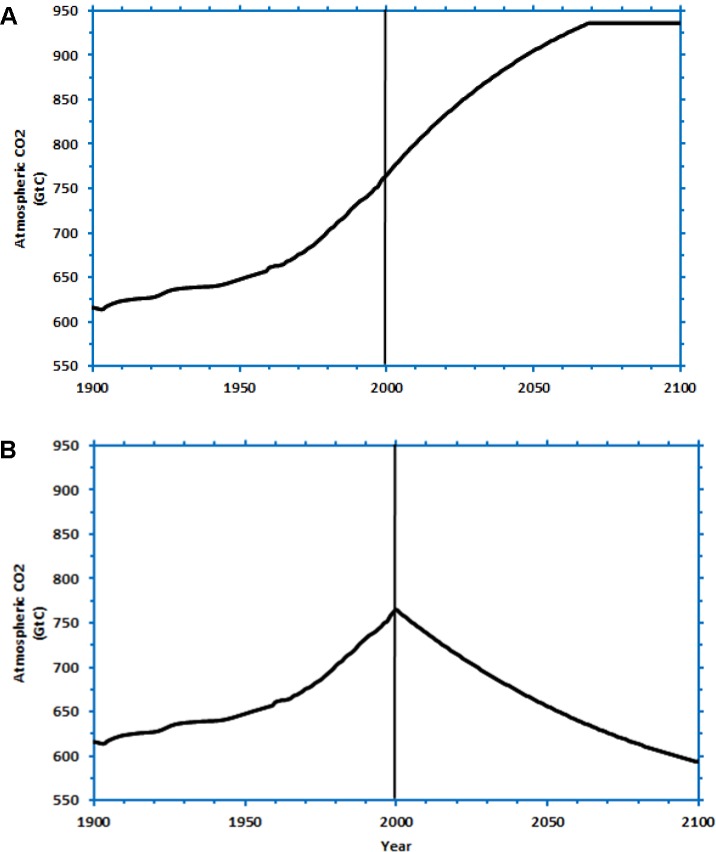
The CO_2_ concentration trajectory given to participants in the DCCS-Structure condition. **(A)** The increasing CO_2_ concentration trajectory, where stabilization occurs in year 2070 (DCCS-2070; CS-2100-Inc). **(B)** The decreasing CO_2_ concentration trajectory, where stabilization occurs in the year 2100 (CS-2100-Dec).

Structural features refer to how emissions and absorptions cause a change in CO_2_ concentration under different CO_2_ concentration scenarios in DCCS. In the DCCS-Structure condition, participants first performed in two different climate problems presented randomly in DCCS. Each problem provided a different CO_2_ stabilization trajectory, where CO_2_ concentration increased from 765GtC in 2000 to stabilize at 936GtC by 2100 or a year before. In one of these DCCS problems, the stabilization occurred in year 2100 (**Figure 1**’s problem; DCCS-2100). In the other problem, the stabilization at 936GtC occurred much earlier in years 2070 (DCCS-2070), respectively, and the 936GtC value was maintained till the end year 2100 (see **Figure 4A** for the shape of the CO_2_ concentration curve). In each of the two DCCS problems, participants were asked to control the CO_2_ concentration to the stabilization trajectory over a 100-year period by making emission and absorption decisions every 5 years, repeatedly. Once participants completed 2-rounds in DCCS, they were transferred to the CS task immediately where participants attempted two problems presented in a random order. One of these two problems were **Figure 1**’s climate problem (CS-2100-Inc; i.e., like one of the problems in the DCCS training), and the other problem was **Figure 4B**’s climate problem (CS-2100-Dec; i.e., different from all problems in the DCCS training). In both CS problems, participants needed to sketch the shape of CO_2_ emissions and absorptions that corresponded to the CO_2_ concentration stabilization scenario. The CS-Structure condition contained the same two problems in the CS task of the DCCS-Structure condition and did not include training in DCCS. In the CS-Structure condition, participants played an unrelated Tetris game before performing the CS task for a duration that equaled the time taken to finish 2-rounds of DCCS performance in the DCCS-Structure condition.

The CS-2100-Inc, CS-2100-Dec, CS-Temp, and CS-Climate conditions formed the control groups in the experiment. The DCCS-2070, DCCS-2100, DCCS-Temp, and DCCS-Gluc formed the training groups in the experiment. The CS-2100-Inc (DCCS), CS-2100-Dec (DCCS), CS-Temp (DCCS), and CS-Climate (DCCS) formed the test groups in the experiment.

The dependent variables were the proportion of participants relying on correlation heuristic and the proportion of participants committing violation of mass balance. A participant relied on correlation heuristic when the correlation coefficient between CO_2_ emissions and CO_2_ concentration during the period 2000–2100 was greater than or equal to 0.8. A participant committed violation of mass balance for the increasing trajectory stabilizing in 2100 (2070), if CO_2_ emissions were less than CO_2_ absorptions before year 2100 (2070) or CO_2_ emissions were not within ± 0.5GtC of CO_2_ absorptions in 2100 (2070 and beyond). A participant committed violation of mass balance for the decreasing trajectory stabilizing in 2100, if CO_2_ emissions were greater than CO_2_ absorptions before year 2100 or CO_2_ emissions were not within ± 0.5GtC of CO_2_ absorptions in 2100. Because of heterogeneity in surface or structural features in DCCS, we expected participants to possess fewer correlation heuristic and violation of mass balance misconceptions in CS conditions following DCCS compared to CS conditions without DCCS exposure. We used an alpha level of 0.05 and a power of 0.80 for our statistical analyses. The dataset for the experiment has been provided as part of **Supplementary Data Sheet S1**.

#### Procedure

Participants were randomly assigned to different conditions and given instructions about the study. Participants were told about the goal that they had to achieve and they could ask clarification questions, if any, before beginning their experiment. In the DCCS-Surface and DCCS-Structure conditions, participants first performed 2-rounds in DCCS on a desktop computer and then they were transferred to CS tasks, where the CS tasks were given using a pencil-and-paper format. However, in the CS-Surface and CS-Structure conditions, participants first performed an unrelated Tetris task and then they were immediately transferred to CS tasks, which were given using a pencil-and-paper format. In the CS task, participants had to sketch CO_2_ emissions and absorptions corresponding to the given CO_2_ concentration trajectory. On completion of the CS task, participants were thanked and paid for their participation.

### Results

#### Correlation Heuristic

**FIGURE 5:**
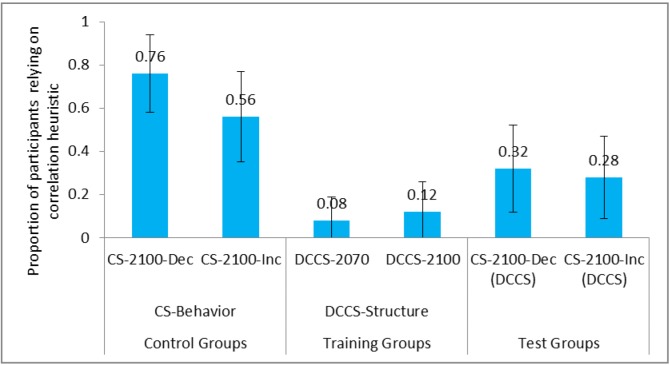
Proportion of participants relying on correlation heuristic in CS tasks and DCCS in CS-Structure and DCCS-Structure conditions. The CS-2100-Dec (DCCS) task and CS-2100-Inc (DCCS) task refer to CS tasks following the DCCS performance in the DCCS-Structure condition. The error bars represent 95% confidence interval around the point estimate.

We compared the correlation heuristic reliance between control groups and test groups in the structure conditions. **Figure 5** shows the proportion of participants relying on correlation heuristic in CS tasks and DCCS in the DCCS-Structure and CS-Structure conditions. Furthermore, **Table 1** shows the comparison of different conditions and the associated inferential statistics for correlation heuristic reliance. As seen in **Table 1**, the reliance on correlation heuristic was statistically smaller in CS-2100-Dec (DCCS) condition compared to CS-2100-Dec condition. Likewise, the reliance on correlation heuristic was statistically smaller in CS-2100-Inc (DCCS) condition compared to CS-2100-Inc condition. Furthermore, the reliance was similar in CS-2100-Dec and CS-2100-Inc conditions. Similarly, the reliance on correlation heuristic was similar in CS-2100-Dec (DCCS) task and CS-2100-Inc (DCCS) condition.

**Table 1:** Comparison of different conditions involving correlation heuristic reliance among participants.

Condition comparisons	Statistical inference		
	χ^2^ (1)	*p*	φ
CS-2100-Dec (DCCS) (0.32) < CS-2100-Dec (0.76)	9.74	<0.001	0.44
CS-2100-Inc (DCCS) (0.28) < CS-2100-Inc (0.56)	4.02	0.04	0.28
CS-2100-Dec (0.76) ∼ CS-2100-Inc (0.56)	2.23	0.14	0.21
CS-2100-Dec (DCCS) (0.28) ∼ CS-2100-Inc (DCCS) (0.32)	0.09	0.76	0.04
CS-Temp (DCCS) (0.24) < CS-Temp (0.96)	27.00	<0.001	0.73
CS-Climate (DCCS) (0.44) < CS-Climate (0.92)	13.24	<0.001	0.51
CS-Temp (0.96) ∼ CS-Climate (0.92)	0.36	0.55	0.08
CS-Temp (DCCS) (0.24) ∼ CS-Climate (DCCS) (0.44)	2.23	0.14	0.21
CS-2100-Inc (0.56) < CS-Climate (0.92)	8.42	<0.001	0.41
CS-2100-Inc (DCCS) (0.28) ∼ CS-Climate (DCCS) (0.44)	1.39	0.24	0.17

*The number in the bracket represents the proportion of participants relying on correlation heuristic. The symbol ∼ indicates that the proportions in two conditions were similar to each other.*

**FIGURE 6:**
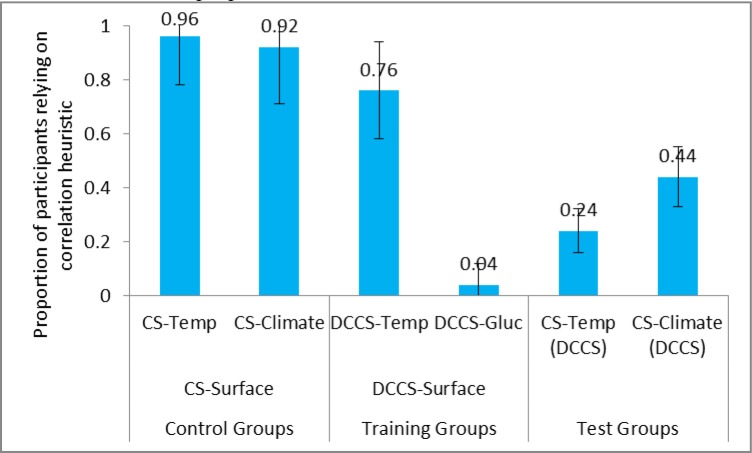
Proportion of participants relying on correlation heuristic in surface conditions. The CS-Temp (DCCS) and CS-Climate (DCCS) refer to CS tasks following the DCCS performance in the DCCS-Surface condition. The error bars represent 95% confidence interval around the point estimate.

Next, we compared the correlation heuristic reliance between the control group and the test group in the surface conditions. **Figure 6** shows the proportion of participants relying on correlation heuristic in CS tasks and DCCS in the DCCS-Surface and CS-Surface conditions. As seen in **Table 1**, the reliance on correlation heuristic was statistically smaller in CS-Temp (DCCS) condition compared to CS-Temp condition. Likewise, reliance was statistically smaller in CS-Climate (DCCS) condition compared to CS-Climate condition. Furthermore, the reliance on correlation heuristic was similar in CS-Temp condition and CS-Climate condition. Similarly, reliance on correlation heuristic was similar in CS-Climate (DCCS) condition and CS-Temp (DCCS) condition.

Last, we compared the correlation heuristic reliance between control groups and test groups across the surface and structure conditions. The reliance on correlation heuristic was statistically smaller in CS-2100-Inc condition compared to CS-Climate condition. However, the reliance on correlation heuristic was similar in CS-2100-Inc (DCCS) condition compared to CS-Climate (DCCS) condition.

Overall, in agreement with our expectations, the proportion of participants relying on correlation heuristic was statistically smaller in DCCS-Structure and DCCS-Surface conditions compared to CS-Structure and CS-Surface conditions, respectively. Also, the correlation heuristic proportions were similar in the CS-2100-Inc (DCCS) and CS-Climate (DCCS) conditions. This latter finding suggested that both the structure and surface features were similar in their ability to reduce people’s correlation heuristic misconceptions.

#### Violation of Mass Balance

**FIGURE 7:**
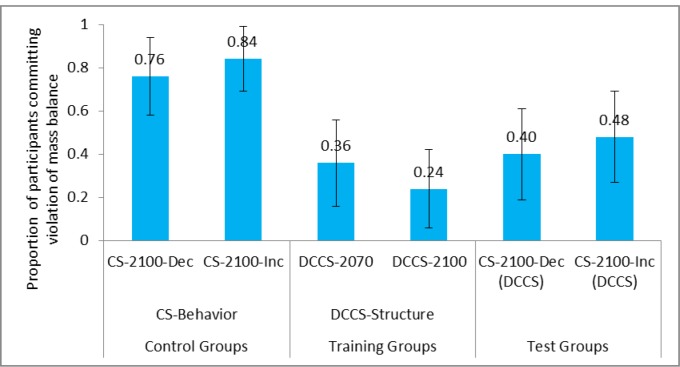
Proportion of participants committing violation of mass balance in CS tasks and DCCS in CS-Structure and DCCS-Structure conditions. The CS-2100-Dec (DCCS) task and CS-2100-Inc (DCCS) task refer to CS tasks following the DCCS performance in the DCCS-Structure condition. The error bars represent 95% confidence interval around the point estimate.

We compared the proportion of participants commiting violation of mass balance between control groups and test groups across the structure conditions. **Figure 7** shows the proportion of participants committing violation of mass balance in CS tasks and DCCS in the DCCS-Structure and CS-Structure conditions. Furthermore, **Table 2** shows the comparison of different conditions and the associated inferential statistics for mass balance violation. As seen in **Table 2**, the proportion of violation of mass balance was statistically smaller in the CS-2100-Dec (DCCS) condition compared to the CS-2100-Dec condition. Furthermore, the proportion of violation of mass balance was statistically smaller in CS-2100-Inc (DCCS) condition compared to CS-2100-Inc condition. The proportion of violation of mass balance was similar in CS-2100-Inc (DCCS) condition and CS-2100-Dec (DCCS) condition. Similarly, the proportion of violation of mass balance was similar in CS-2100-Inc condition and CS-2100-Dec condition.

**Table 2:** Comparison of different conditions involving violation of mass balance among participants.

Condition comparisons	Statistical inference		
	χ^2^ (1)	*p*	φ
CS-2100-Dec (DCCS) (0.40) < CS-2100-Dec (0.76)	6.65	<0.001	0.36
CS-2100-Inc (DCCS) (0.48) < CS-2100-Inc (0.84)	7.22	<0.001	0.38
CS-2100-Dec (0.76) ∼ CS-2100-Inc (0.84)	0.50	0.48	0.10
CS-2100-Dec (DCCS) (0.40) ∼ CS-2100-Inc (DCCS) (0.48)	0.33	0.57	0.08
CS-Temp (DCCS) (0.20) < CS-Temp (0.92)	26.30	<0.001	0.72
CS-Climate (DCCS) (0.24) < CS-Climate (0.88)	20.78	<0.001	0.64
CS-Temp (0.92) ∼ CS-Climate (0.88)	0.22	0.64	0.07
CS-Temp (DCCS) (0.20) ∼ CS-Climate (DCCS) (0.24)	0.12	0.74	0.05
CS-2100-Inc (0.84) ∼ CS-Climate (0.88)	0.17	0.68	0.06
CS-2100-Inc (DCCS) (0.48) ∼ CS-Climate (DCCS) (0.24)	3.13	0.07	0.25

*The number in the bracket represents the proportion of participants committing violation of mass balance. The symbol ∼ indicates that the proportions in two conditions were similar to each other.*

**FIGURE 8:**
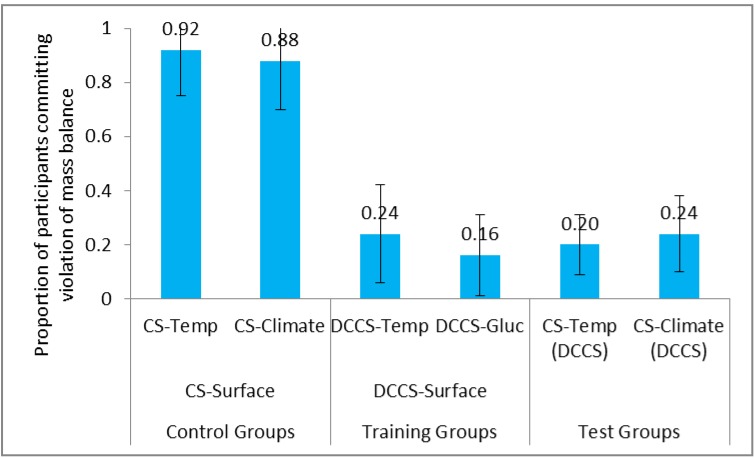
Proportion of participants committing violation of mass balance in surface conditions. The CS-Temp (DCCS) and CS-Climate (DCCS) refer to CS tasks following the DCCS performance in the DCCS-Surface condition. The error bars represent 95% confidence interval around the point estimate.

Next, we compared the violation of mass balance between control groups and test groups across the surface conditions. **Figure 8** shows the proportion of participants committing violation of mass balance in CS tasks and DCCS in the DCCS-Surface and CS-Surface conditions. As seen in **Table 2**, the proportion of violation of mass balance was statistically smaller in CS-Temp (DCCS) condition compared to CS-Temp condition. Likewise, the proportion of violation of mass balance was statistically smaller in CS-Climate (DCCS) condition compared to CS-Climate condition. Furthermore, the proportion of violation of mass balance was similar in the CS-Temp condition and CS-Climate condition. Similarly, the proportion of violation of mass balance was similar in CS-Climate (DCCS) condition and CS-Temp (DCCS) condition.

Last, we compared the violation of mass balance across the surface and structure conditions. The proportion of violation of mass balance was similar in CS-2100-Inc condition compared to CS-Climate condition. Similarly, the proportion of violation of mass balance was similar in CS-2100-Inc (DCCS) condition compared to CS-Climate (DCCS) condition. This latter finding suggested that both the structure and surface features were similar in their ability to reduce people’s violation of mass balance misconceptions.

Thus, overall, the experience gained in DCCS helped participants to reduce mass balance violations. Furthermore, the violation of mass balance reduction helped participants to perform better in the following CS task in the DCCS conditions compared to that in the CS conditions in both structure and surface condition.

### Discussion

The comparison of the problems in the CS tasks of DCCS condition and CS condition allowed us to measure the effectiveness of the surface or structural heterogeneity in reducing correlation heuristic and violation of mass balance misconceptions. In both the surface and structure conditions, misconceptions related to correlation heuristic and violation of mass balance reduced significantly in the CS tasks following DCCS compared to CS tasks without exposure in DCCS.

First, we found that when we changed the problem’s structural features between DCCS and the following CS task (i.e., change the way CO_2_ emissions and absorptions affect the CO_2_ concentration), misconceptions reduce significantly in the CS task post DCCS performance. This finding agrees with recent research that showed that structural knowledge helped people reduce their correlation heuristic and violation of mass balance misconceptions in both cases when problems encountered in the CS task are structurally similar or different compared to those presented in DCCS (Kumar and Dutt, unpublished). In our study, when people attempt to follow different trajectories of CO_2_ concentration in DCCS, then this exposure to heterogeneous system dynamics likely enables them to learn that the CO_2_ concentration increases when CO_2_ emissions are greater than CO_2_ absorptions, decreases when CO_2_ emissions are smaller than CO_2_ absorptions, and stabilizes when CO_2_ emissions equal CO_2_ absorptions.

Second, we found that when we changed the problem’s surface features in DCCS, then misconceptions also reduced significantly in the CS task post DCCS performance. One likely reason for this finding is that people get to learn via DCCS that the same system dynamics applies across different dimensions and cover stories. Thus, they could transfer this learning in CS tasks post DCCS performance.

Overall, our results agree with the procedural reinstatement principle (Healy et al., 2005), where we found improved performance when problems in the CS task were similar in structure or surface features to those that were learned during DCCS training (prior to the CS task). Also, our results agree with the heterogeneity of practice hypothesis (Gonzalez and Madhavan, 2011), where we found that problems with surface or structural training during DCCS were able to produce more effective transfer of knowledge and improved performance in the CS task.

There were some differences in the curve shapes and cover stories used between tasks across surface and structure conditions. Thus, we could not compare all tasks across these conditions. However, upon comparing tasks that were similar in their curve shapes and cover stories used, we did find a similar reduction in correlation heuristic and violation of mass balance misconceptions across the surface and structure conditions. Overall, these results indicate that both surface and structural heterogeneity is equally powerful in reducing people’s stock-flow misconceptions.

Although the problems used in the current experiment created learning of structural and surface features for participants, there may be other ways of creating effective training conditions. However, as part of future work we would like to compare structure and surface heterogeneity with homogenous conditions. For example, one other way learning could be influenced during DCCS training is by varying the difficulty level of problems in DCCS. The problem difficulty could be varied in DCCS based upon the shape of CO_2_ concentration trajectory that participants are asked to follow in DCCS. The next experiment explores the effects of problem difficulty in reducing correlation heuristic and violation of mass balance misconceptions.

## Experiment 2: Effect of Difficulty of Problems in Reducing Stock-Flow Misconceptions

Another way in which training conditions might differ is by the difficulty of problems encountered. For example, school children may be trained on simple and difficult problems in the classroom to prepare them for different problems in their exam. According to the difficulty hypothesis (Schneider et al., 2002; Young et al., 2011), transfer performance in the CS task should improve when training is conducted using difficult climate problems in DCCS compared to simple problems. However, it is also possible that due to the predictions from cognitive load theory (Sweller, 1994; De Jong, 2010), difficult training problems in DCCS may not lead to reductions in stock-flow misconceptions compared to simple training problems.

### Methods

#### Participants

Participants were recruited through an email advertisement for a climate-study at Indian Institute of Technology, Mandi, India. This study was carried out in accordance with the recommendations of Ethics Committee at Indian Institute of Technology Mandi with a written informed consent from all participants. Participation was voluntary and all participants gave written informed consent before starting their study. There were 90 participants in all (78 males and 12 females). Ages ranged from 18 to 25 years (average = 23 years; *SD* = 1.4 years). All participants were from Science, Technology, Engineering, and Mathematics backgrounds (88% undergraduate, 9% masters, and 3% doctoral). They were randomly assigned to one of the experimental conditions involving DCCS and CS tasks. Participants were paid a flat fee of INR 50 (approximately 0.9 USD) for their participation after they completed the study.

#### Experimental Design

**FIGURE 9:**
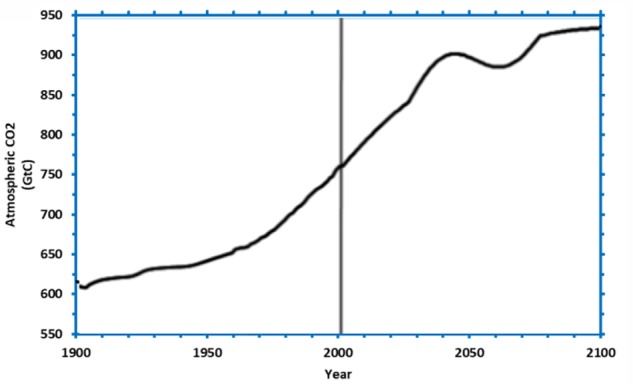
The CO_2_ concentration stabilization trajectory to be used as the difficult problem.

Participants were randomly assigned to one of the following three between-subjects conditions (*N* = 30 in each condition): DCCS-Difficult, DCCS-Easy and CS. In the DCCS-Difficult and DCCS-Easy conditions, participants first performed 1-round in DCCS and were immediately transferred to the CS task. In the DCCS-Easy and DCCS-Difficult conditions, in DCCS, participants controlled the CO_2_ concentration to the stabilization trajectory in each round by making inflow and outflow decisions every 5 time periods repeatedly. In the DCCS-Easy condition, the DCCS used **Figure 1**’s problem. However, in the DCCS-Difficult condition, the DCCS used **Figure 9**’s problem. The shape of CO_2_ concentration scenario in **Figure 9**’s problem was more complex compared to that in **Figure 1**’s problem (although the CO_2_ concentration in both problems had about the same values and direction of movement over time). The complexity of the concentration curve made **Figure 9**’s problem more difficult compared to **Figure 1**’s problem. After participants finished performing in DCCS, they were transferred to a different problem in the CS task. In the CS condition, however, participants played an unrelated Tetris task for the average time it took to complete 1-round in the DCCS task (in the conditions involving DCCS) and were transferred to the CS task immediately. In CS tasks across all conditions, participants attempted the problem shown in **Figure 4B**, where they sketched the shape of CO_2_ emissions and absorptions that corresponded to a decreasing CO_2_ concentration stabilization trajectory between 2001 and 2100. In this experiment, the CS condition formed the control group, the DCCS-Easy and DCCS-Difficult conditions formed the training groups, and the CS (DCCS-Easy) and CS (DCCS-Difficult) formed the test groups.

The dependent variables were the proportion of participants relying on correlation heuristic and the proportion of participants committing violation of mass balance. The coding used to classify participants as relying on correlation heuristic and committing violation of mass balance across the control, training, and test groups was the same as that used in Experiment 1. The alpha and power levels were same as reported in experiment 1. The dataset for the experiment has been provided as part of **Supplementary Data Sheet S1**.

#### Procedure

Participants were randomly assigned to different conditions and given instructions about the study. Participants were told about the goal that they had to achieve and they could ask clarification questions, if any, before beginning their experiment. In the DCCS-Easy and DCCS-Difficult conditions, participants performed 1-round in DCCS on a desktop computer and then they were transferred to CS tasks, where the CS tasks were given using a pencil-and-paper format. However, in the CS condition, participants first performed an unrelated Tetris task and then they were immediately transferred to the CS task, which was given using a pencil-and-paper format. In the CS task, participants had to sketch CO_2_ emissions and absorptions corresponding to the CO_2_ concentration trajectory. On completion of the CS task, participants were thanked and paid for their participation.

### Results

#### Correlation Heuristic

**FIGURE 10:**
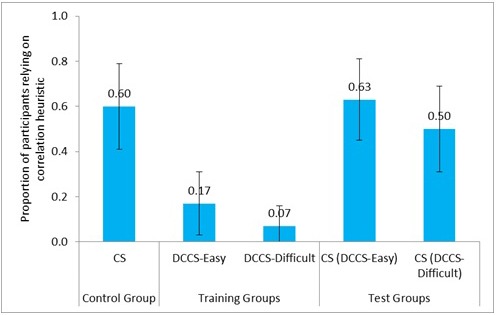
Proportion of participants relying on correlation heuristic in three conditions: DCCS-Easy, DCCS-Difficult, and CS conditions. The CS (DCCS-Easy) task and CS (DCCS-Difficult) task refer to CS tasks following the DCCS performance in the DCCS-Easy and DCCS-Difficult conditions. The error bars represent 95% confidence interval around the point estimate.

We compared the correlation heuristic reliance between the control group and the test groups across the easy and difficult conditions. **Figure 10** shows the proportion of participants relying on correlation heuristic in CS tasks and DCCS in the DCCS-Easy, DCCS-Difficult, and CS conditions. Furthermore, **Table 3** shows the comparison of different conditions and the associated inferential statistics for correlation heuristic reliance. As seen in **Table 3**, the reliance on correlation heuristic was similar across the CS tasks in the CS condition and the DCCS-Difficult condition. Similarly, the reliance on correlation heuristic was similar across the CS tasks in the CS condition and the DCCS-Easy condition. Furthermore, the proportion of participants relying on correlation heuristic was similar across the CS tasks in the DCCS-Easy condition and the DCCS-Difficult condition.

**Table 3:** Comparison of different conditions involving correlation heuristic reliance among participants.

Condition comparisons	Statistical inference		
	χ^2^ (1)	*p*	φ
CS (0.60) ∼ CS (DCCS-Difficult) (0.50)	0.61	0.44	0.10
CS (0.60) ∼ CS (DCCS-Easy) (0.63)	0.07	0.79	0.03
CS (DCCS-Easy) (0.63) ∼ CS (DCCS-Difficult) (0.50)	1.09	0.29	0.13

*The number in the bracket represents the proportion of participants relying on correlation heuristic. The symbol ∼ indicates that the proportions in two conditions were similar to each other.*

#### Violation of Mass Balance

**FIGURE 11:**
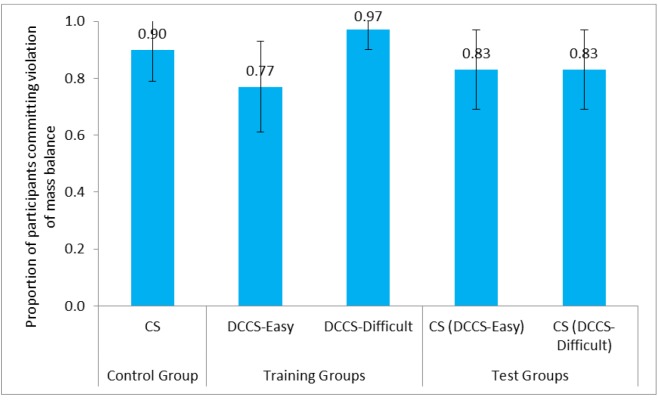
Proportion of participants committing violation of mass balance in three conditions: DCCS-Easy, DCCS-Difficult, and CS conditions. The CS (DCCS-Easy) task and CS (DCCS-Difficult) task refer to CS tasks following the DCCS performance in the DCCS-Easy and DCCS-Difficult conditions. The error bars represent 95% confidence interval around the point estimate.

We compared the committing of violation of mass balance between the control group and the test groups across the easy and difficult conditions. **Figure 11** shows the proportion of participants committing violation of mass balance in CS tasks and DCCS in DCCS-Easy, DCCS-Difficult, and CS conditions. **Table 4** shows the comparison of different conditions and the associated inferential statistics for mass balance violation. As seen in **Table 4**, results indicated that the proportion of violation of mass balance was similar across the CS tasks in the CS condition and the DCCS-Difficult condition. Furthermore, the proportion of violation of mass balance was similar across the CS tasks in the CS condition and the DCCS-Easy condition. Likewise, the proportion of violation of mass balance was similar across the CS tasks of the DCCS-Easy condition and the DCCS-Difficult conditions.

**Table 4:** Comparison of different conditions involving violation of mass balance among participants.

Condition comparisons	Statistical inference		
	χ^2^ (1)	*p*	φ
CS (0.90) ∼ CS (DCCS-Difficult) (0.83)	0.58	0.45	0.10
CS (0.90) ∼ CS (DCCS-Easy) (0.83)	0.58	0.45	0.10
CS (DCCS-Easy) (0.83) ∼ CS (DCCS-Difficult) (0.83)	0.00	1.00	0.00

*The number in the bracket represents the proportion of participants committing violation of mass balance. The symbol ∼ indicates that the proportions in two conditions were similar to each other.*

Overall, in agreement with the expectations from cognitive load theory, the proportion of participants relying on CH and committing violation of mass balance were similar in the CS tasks of the DCCS-Difficult condition and the DCCS-Easy condition.

### Discussion

Variation in problem difficulty could be another way of enabling learning among people that reduces their stock-flow misconceptions. In this experiment, we varied problem difficulty in terms of the shape of the CO_2_ concentration trajectory: smooth (simple) or curvilinear (difficult). We found that people could not reduce their correlation heuristic misconceptions after exposure to difficult climate problems in DCCS compared to those who were either not provided DCCS training or were only exposed to easy climate problems in DCCS. Similarly, the same intervention did not reduce the violation of mass balance misconceptions: The committing of violation of mass balance remained the same after DCCS training (among both easy and difficult problems) in the CS task compared to conditions where the CS task was given without exposure in DCCS. The lack of reduction in correlation heuristic and violation of mass balance misconceptions could be attributed to cognitive load theory (Simon, 1991; Sweller, 1994; De Jong, 2010). As per cognitive load theory, it is possible that the processing of different elements like emission, absorption, and the curvilinear concentration in the DCCS task required too much working memory capacity. Due to the cognitive overload and bounded memory capacity, participants were not able to reduce their correlation heuristic and violation of mass balance misconceptions.

Our results in this experiment did not agree with the expectations from the difficulty hypothesis (Schneider et al., 2002; Young et al., 2011). Perhaps, the shape of the difficult CO_2_ concentration trajectory was not difficult enough in making people learn reduce their stock-flow misconceptions. Although we can only speculate currently, a more challenging CO_2_ concentration trajectory in DCCS that gives exposure to people about increase, decrease, and stabilization of accumulation may help reduce people’s misconceptions.

Beyond testing the difficulty of problems and their effectiveness in DCCS, another way for reducing correlation heuristic and violation of mass balance misconceptions could be by using simulation tools as side-by-side decision aids (e.g., a computer or calculator). The focus of the next experiment is to evaluate how DCCS could be used as a side-by-side decision aid in reducing stock-flow misconceptions.

## Experiment 3: Effect of Decision Aids in Reducing Stock-Flow Misconceptions

There are numerous situations in life like during schooling when students make use of decision aids (e.g., computers and calculators) to assist them in solving complex mathematical problems. Similarly, climate-scientists and climate-policymakers are likely to use decision aids (e.g., simulation tools) while formulating future greenhouse gas emission policies. For example, to evaluate the effects of future emission policies on the CO_2_ concentrations and global temperatures we may need to rely upon decision aids. In simple descriptive binary-choice decision tasks, when participants are provided with experiential decision aids, they tend to rely on the experience gained in these aids in making descriptive decisions and improving their decision making (Jessup et al., 2008; Camilleri and Newell, 2011; Lejarraga and Gonzalez, 2011). The aim of this experiment is to evaluate the effectiveness of decision aids in reducing people’s misconceptions when they have at their disposal an aid that simulates future CO_2_ concentrations by assuming different CO_2_ emission policies.

### Methods

#### Participants

Participants were recruited through an email advertisement for a climate-study at Indian Institute of Technology Mandi, India. This study was carried out in accordance with the recommendations of Ethics Committee at Indian Institute of Technology Mandi with a written informed consent from all participants. Participation was voluntary and all participants gave written informed consent before starting their study. There were 60 participants in all (52 males and 08 females). Ages ranged from 18 to 26 years (average = 22 years; *SD* = 1.5 years). All participants were from Science, Technology, Engineering, and Mathematics backgrounds (85% undergraduate, 12% masters, and 3% doctoral). They were randomly assigned to one of the conditions involving DCCS and CS tasks. Participants were paid a flat fee of INR 50 (approximately 0.9 USD) for their participation after they completed the study.

#### Experimental Design

Participants were randomly assigned to one of two between-subjects conditions (*N* = 30 in each condition): Aid and No-aid. In the Aid condition, participants could use DCCS side-by-side as a decision aid while sketching the CO_2_ emissions and absorptions in the CS task; however, in the No-aid condition, participants only sketched the CO_2_ emissions and absorptions in the CS task and they did not use DCCS. In the Aid condition, participants could use DCCS anytime to enter 10-yearly emission and absorption values over a period of 100 years (i.e., a total of 10 values for each of the emissions and absorptions) and simulate the resulting CO_2_ concentration. The DCCS simulated the entered emissions and absorptions rapidly within 1 to 2 seconds. Participants could then reset DCCS to the year 2000 and simulate a different set of emission and absorption values. In the Aid condition, participants could use DCCS as many times as they wanted to before they sketched the CO_2_ emissions and absorptions in the CS task. Also, the number of times participants used the DCCS as a decision aid was recorded in the Aid condition. In the No-aid condition, participants were asked to play a Tetris game for an amount time that equaled the time that participants took to use DCCS in the Aid condition. The No-aid condition formed the control group and the Aid condition formed the test group.

The dependent variables were the proportion of participants relying on correlation heuristic and the proportion of participants committing violation of mass balance. In the Aid condition, the correlation heuristic and violation of mass balance misconceptions were analyzed in DCCS by using the averaged emission and absorption trajectory, where the average was computed across the number of times DCCS was used as a decision aid. In both Aid and No-aid conditions, participants attempted a single problem in the CS task and that was the one shown in **Figure 4B**. The coding used to classify participants as relying on correlation heuristic and committing violation of mass balance across the control and test groups was the same as that used in Experiment 1. Because of the presence of DCCS, we expected smaller proportions of correlation heuristic and violation of mass balance in the CS task in Aid condition compared to the No-aid condition. The alpha and power levels were the same as reported in experiment 1. The dataset for the experiment has been provided as part of **Supplementary Data Sheet S1**.

#### Procedure

Participants were randomly assigned to different conditions and given instructions about the study. Participants were told about the goal in the CS task: to sketch the CO_2_ emission and absorption trajectories that would correspond to the CO_2_ concentration trajectory. Participants could ask clarification questions, if any, before starting their study. In the Aid condition, participants were encouraged to use DCCS as a decision aid side-by-side the CS task. However, in the No-aid condition, participants first performed in the unrelated Tetris task and then they were immediately transferred to the CS task. On completion of the CS task, participants were paid for their participation.

### Results

First, we analyzed the number of times DCCS was used as a decision aid in the Aid condition. Results revealed that participants used DCCS between 1 time and 7 times in the Aid condition (average = 3 times, *SD* = 1.4 times).

#### Correlation Heuristic

**FIGURE 12:**
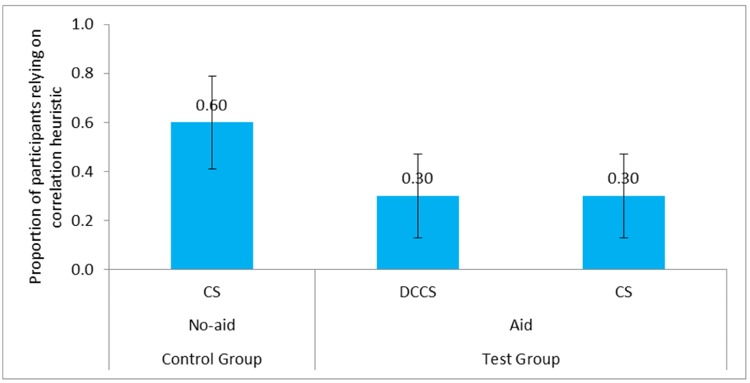
Proportion of participants relying on correlation heuristic in the Aid and No-aid conditions. The error bars represent 95% confidence interval around the point estimate.

We compared the correlation heuristic reliance between the CS tasks across the Aid and No-aid conditions. **Figure 12** shows the proportion of participants relying on correlation heuristic in the Aid and No-aid conditions. Results revealed that reliance on correlation heuristic was statistically smaller in the CS task of Aid condition compared to the CS task of No-aid condition [0.30 < 0.60, χ^2^ (1) = 5.46, *p* = 0.02, φ = 0.30]. The proportion of participants relying on correlation heuristic in DCCS was close to 0.30. The correlation between the number of times DCCS was used and reliance on correlation heuristic in the CS task was small and insignificant (*r* = 0.14, *p* = 0.41).

#### Violation of Mass Balance

**FIGURE 13:**
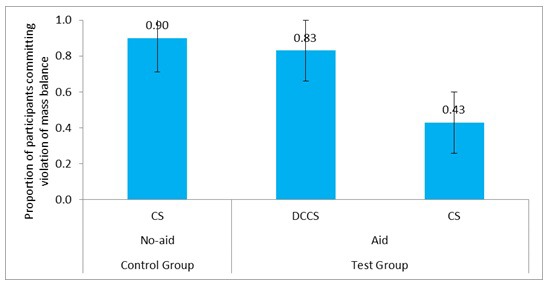
Proportion of participants committing violation of mass balance in the Aid and No-Aid conditions. The error bar represents the 95% confidence interval around the point estimate.

We compared the committing of violation of mass balance between the CS tasks across the Aid and No-aid conditions. **Figure 13** shows the proportion of participants committing violation of mass balance in the Aid and No-aid conditions. Results indicated that violation of mass balance was statistically smaller in the CS task of Aid condition compared to the CS task of No-aid condition [0.43 < 0.90, χ^2^ (1) = 14.70, *p* = 0.00, φ = 0.49]. The proportion of participants committing violation of mass balance in DCCS was close to 0.85. The correlation between the number of times DCCS was used and committing of violation of mass balance in the CS task was small and insignificant (*r* = 0.08, *p* = 0.62).

Overall, in agreement with our expectations, the proportion of participants relying on correlation heuristic and committing violation of mass balance was statistically smaller in Aid condition compared to No-aid condition.

### Discussion

Simulations tools may provide effective side-by-side decision aids that enable people to reduce their stock-flow misconceptions. Results revealed that DCCS served as an effective side-by-side decision aid and enabled people to reduce their correlation heuristic and violation of mass balance misconceptions compared to those conditions where DCCS was not present.

One likely reason for the effectiveness of DCCS as a decision aid could be that DCCS enables people to try different scenarios related to how CO_2_ emissions and absorptions influence the trajectory of CO_2_ concentration (Dörner, 1996; Cronin et al., 2009; Dutt, 2011). Thus, people could use DCCS to try different CO_2_ emissions and absorptions values and observe their effect on the resulting CO_2_ concentration trajectories. This trial-and-error learning in DCCS is consistent with literature on experienced-based decisions (Jessup et al., 2008; Camilleri and Newell, 2011; Lejarraga and Gonzalez, 2011). For example, according to Jessup et al. (2008), when participants are provided with experiential decision problems, they tend to rely on the experience gained in these problems in making decisions and improve their decision making. Similarly, the experience gained in DCCS enables participants to improve their decision-making in the CS task.

Furthermore, in our results, participants used DCCS between 1 time and 7 times before while attempting the CS task. This use of DCCS agrees with that reported in literature (Hertwig et al., 2004; Cronin et al., 2009). For example, Cronin et al. (2009) gave a stock-flow problem where participants needed to determine the maximum and minimum stock levels across multiple attempts. In each attempt, participants wrote answers to stock questions and they were given feedback on whether their answers were correct or incorrect. According to Cronin et al. (2009), due to the correct-incorrect feedback, more than 70% of the participants were able to answer the stock questions correctly by the fifth attempt (i.e., between one and nine attempts). Similarly, in agreement with our results, Hertwig et al. (2004) have shown that people explore different options presented to them about 7 times before choosing an option for real.

## General Discussion

In this paper, we started with the general hypothesis that heterogeneity in surface, structure, and problem difficulty in simulation tools as well as the use of the simulation tools as decision aids will be helpful in reducing public stock-flow misconception about Earth’s climate. Across the first two experiments, we evaluated how the DCCS enables people to reduce their climate misconceptions because of heterogeneity due to surface and structural features as well as problem difficulty. Also, in a third experiment, we evaluated how DCCS as a side-by-side decision aid helps people to reduce their climate misconceptions. Overall, our results could be explained based upon theoretical arguments concerning the heterogeneity of practice hypothesis (Gonzalez and Madhavan, 2011), procedural reinstatement principle (Schneider et al., 2002; Healy et al., 2005; Young et al., 2011), cognitive load theory (Sweller, 1994; De Jong, 2010), and decisions from experience (Jessup et al., 2008; Camilleri and Newell, 2011; Lejarraga and Gonzalez, 2011).

First, our findings suggest that simulation tools for Earth’s climate (like DCCS) are effective in causing learning of both structural features and surface features in problems. In our experiment, people were not given full-information on the formulations connecting emission, absorption, and concentration (Dörner, 1996). These relationships were something that participants had to learn over time while performing in DCCS (Dörner, 1996). Based upon our results, simulation tools like DCCS not only enable people to learn the generality of problems across units and dimensions but also the generality of problems across how inputs and outputs influence the accumulation (Dörner, 1996; Sutton and Barto, 1998; Gonzalez et al., 2003; Dutt and Gonzalez, 2015).

Dutt and Gonzalez (2015) have provided a cognitive account based upon Instance-based Learning Theory (IBLT) on how learning occurs as a dynamic task (like DCCS) due to the focus on process measures and outcome measures. In agreement with Dutt and Gonzalez (2015)’s account, when people come across elements like emission, absorption, and concentration in DCCS, they create instances (or experiences) in their memory. Several experiences get created due to the repeated interaction in DCCS concerning emission, absorption, and concentration values. However, among these instances those instances that allow people to make their CO_2_ concentration come closer to the goal are the ones that likely get reinforced over time. While performing the CS task, people retrieve these reinforced instances from memory to make improved decisions. Thus, people likely use their reinforced knowledge acquired in DCCS to draw correct trajectories of emissions and absorptions corresponding to the different concentration curves.

Furthermore, our results revealed that the use of complex curve shapes in simulation tools (i.e., problem difficulty), however, did not help participants to reduce their stock-flow misconceptions. This result could be explained based upon the additional working memory capacity requirements to process complex interaction of different elements like emissions, absorptions, and concentrations (Dörner, 1996). In agreement with cognitive load theory, as our working memory is bounded (Simon, 1959), people may not be able to process the complex interactions, especially when the concentration curve shapes are complex.

We found that the difficulty hypothesis was unable to account for the findings in the second experiment. One likely reason for this observation could be that the tasks used in our study are different from those that were used for showcasing the difficulty hypothesis (Healy et al., 2005). In literature, the difficulty hypothesis has been showcased using a duration production task in which the dependent measure was reaction time and not the inflow, outflow, and stock. In DCCS, however, the main dependent variables of interest were the inflow, outflow, and stock. Still, another likely reason for the inability of the difficulty hypothesis could be the trajectory of the stock curve used in the difficult condition. It is likely that the stock shapes used in the difficult condition were not difficult enough to cause learning of the underlying relationship between emissions, absorptions, and concentration. Future research should test the learning from complex concentration curves in simulation tools by trying scenarios with concentration curves of different difficulty (Dörner, 1996). Perhaps, stock-flow problems with more challenging CO_2_ accumulation curves would be more likely to help reduce correlation heuristic and violation of mass balance misconceptions.

We also found that the stock-flow misconceptions did not reduce when the concentration curve shape in DCCS was simple compared to when people were not exposed to DCCS at all. Thus, overall, this result disagrees with those reported in the first and second experiment, where performance in DCCS caused people to reduce their stock-flow misconceptions compared to conditions where participants were not exposed to DCCS. One likely reason for the disagreement could be the number of repetitions of DCCS given in the second experiment (equal to one) compared to other experiment (multiple). Although we can only speculate currently, but, perhaps, more repetitions of DCCS in simple and difficult conditions could lead people to reduce their stock-flow misconceptions. This hypothesis needs to be tested as part of future research.

Our findings have important implications for real-world climate education as well as climate policymaking. First, as simulation tools like DCCS likely create both surface and structure learning, they are ideal for educating students from kindergarten to standard 12th about stock-flow problems (Gonzalez and Wong, 2012; Meadows et al., 2016). Thus, the use of simulation tools should be encouraged in schools for learning about Earth’s climate, especially when students are exposed to concepts like the carbon-cycle and climate change.

Third, the use of simulation tools as decision aids should be encouraged for both climate education and policy analyses. Here, simulation tools can be used as a side-by-side decision aid that provides people the ability to test different hypotheses concerning emissions, absorptions, and concentrations. Also, policymakers could use simulation tools like DCCS for climate policy analyses and to evaluate how different CO_2_ emission and absorption trajectories impacts CO_2_ concentrations and global temperatures. One expects improved policy analyses with repeated iterations in simulation tools.

The current investigation on the use of simulation tools has revealed promising results. However, there are several research questions to pursue as part of research in the immediate future. Although different structural and surface training was taken into account, comparison with homogenous condition was not made. As part of future research, we would like to compare different structural and surface heterogeneous condition with homogeneous conditions. For example, it would be interesting to analyze how heterogeneity in structure, surface, and problem difficulty interacts with people’s science education and other demographic variables. Also, how a group of decision-makers (in contrast to single decision-makers) may improve their correlation heuristic and violation of mass balance misconceptions via simulation tools as well as how these groups show learning of structure, surface, and difficulty? Still, how people who improve their understanding of Earth’s climate in problems with a single accumulation (CO_2_ concentration) improve their decision-making in problems with two or more accumulations (e.g., CO_2_ concentration and global temperatures)? It would be interesting to investigate whether it is people’s conscious or unconscious learning that improves due to the use of simulation tools? And, whether people are really learning something about climate change or just learning to use the DCCS tool to complete the CS task?

Prior research has also reported that a part of the stock-flow misconceptions in the CS task could be because of the format of presentation of material concerning emissions, absorptions, and concentration (Fischer et al., 2015). As per Fischer et al. (2015), the use of verbal formats of presentation of stock-flow problems may help reduce some of the stock-flow misconceptions concerning reasoning about stocks. Thus, as part of future research, it would be interesting to test the effectiveness of the heterogeneity in structure, surface, and problem difficulty as well as the extent of learning (conscious or unconscious) in different verbal and non-verbal stock-flow problem formats.

As part of our future work, we would like to answer some of these open-ended questions by involving complex stock-flow problems that vary in their complexity in terms of the number of stock and flows and nature of stock and flows (Frensch and Funke, 2014). Also, how the increasing complexity of stock-flow problems may interact with the format of presentation of stock-flow problems to influence people’s reduction in stock-flow misconceptions (Fischer et al., 2015). Furthermore, one also needs to go deeper to understand the memory processes underlying the learning of structure and surface features in DCCS (Dörner, 1996). Thus, one also needs to evaluate how certain computational models based upon theories of cognition are likely to provide an account of the changes in memory processes in simulation tools (Gonzalez et al., 2003; Gonzalez and Dutt, 2011). We plan to undertake some of these research questions as part of our immediate research on the theme of learning via simulation tools.

## Author Contributions

MK was the research lead who designed the experiments and carried out data collection for this work. VD was the principal investigator who served as a constant guiding light for this work.

## Conflict of Interest Statement

The authors declare that the research was conducted in the absence of any commercial or financial relationships that could be construed as a potential conflict of interest.

## Footnotes

1 A power calculation with alpha level 0.05 and beta level 0.20 revealed a minimum sample size of 22. Thus, sample sizes of more than 22 were adequate for analyses reported in this paper (Faul et al., 2007)

## Supplementary Material

DATA SHEET S1 Experimental data used in experiments 1, 2 and 3.
